# Imaging-based assessment of response to olaparib in platinum-sensitive relapsed ovarian cancer patients

**DOI:** 10.3389/fonc.2025.1546324

**Published:** 2025-06-05

**Authors:** Maria Delgado-Ortet, Vlad Bura, Ionut-Gabriel Funingana, David Hulse, Leonardo Rundo, James D. Brenton, Evis Sala, Lorena Escudero Sanchez

**Affiliations:** ^1^ Department of Radiology, University of Cambridge, Cambridge, United Kingdom; ^2^ Cancer Research UK Cambridge Centre, CRUK and University of Cambridge, Cambridge, United Kingdom; ^3^ Cambridge University Hospitals NHS Foundation Trust, University of Cambridge, Cambridge, United Kingdom; ^4^ Department of Radiology, Clinical Emergency Hospital for Children, Cluj-Napoca, Romania; ^5^ Department of Oncology, University of Cambridge, Cambridge, United Kingdom; ^6^ Cancer Research UK Cambridge Institute, CRUK and University of Cambridge, Cambridge, United Kingdom; ^7^ Department of Information and Electrical Engineering and Applied Mathematics, University of Salerno, Fisciano, Italy; ^8^ Dipartimento Diagnostica per Immagini, Radioterapia Oncologica ed Ematologia, Policlinico Universitario A. Gemelli IRCCS, Rome, Italy; ^9^ Dipartimento di Scienze Radiologiche ed Ematologiche, Universita Cattolica del Sacro Cuore, Rome, Italy

**Keywords:** ovarian cancer, PARP inhibitors, immunotherapy, radiomics, computed tomography

## Abstract

**Background:**

High-grade serous carcinoma is a highly metastatic disease with a limited longterm disease control from systemic anti-cancer treatment, for which the radiological treatment response assessment metrics are imprecise. In this work, we developed noninvasive imagingbased measurements of spatial and longitudinal heterogeneity in a retrospective analysis of a phase 2 non-randomized study of germline BRCA1/BRCA2 mutated (gBRCAm) ovarian cancer patients treated with combination of PARP inhibitors (PARPi) and immune checkpoint inhibitors (ICIs).

**Methods:**

Lesions identified in CT images at baseline, week 4 (after PARPi only) and week 12 (after 8 weeks of PARPi + ICIs) were manually segmented. Anatomical networks of the metastatic sites were constructed to represent patterns of disease distribution. Volume and first-order radiomic features were computed and compared to different assessments of treatment response.

**Results:**

The average number of edges per patient in the anatomical networks and total volumetric burden decreased with treatment were measured, differentiating between responders and nonresponders. Changes in volume at week 4 provided better indication of long-term response than the default RECIST assessment at the same time-point. Significant differences were also found between responders and non-responders in the first-order radiomic feature Energy.

**Conclusions:**

In this feasibility study, we have demonstrated that noninvasive image-based analysis can identify quantitative imaging features associated with the response to the combination of PARPi and ICIs. These can be used to identify markers of response to ICIs from negative trials of a disease with limited response to ICIs.

## 1 Introduction

High-grade serous carcinoma (HGSC) remains a major clinical problem with a high mortality index compared to other cancers Lisio et al. (1). It is the most prevalent and aggressive subtype of epithelial ovarian cancer with approximately 7,500 women diagnosed every year in the UK CRUK (2). HGSC is typically diagnosed at an advanced stage with multi-site metastatic disease due to its late presentation with nonspecific symptoms Labidi-Galy et al. (3). The poor prognosis and the limited long-term disease control from systemic anti-cancer treatment for HGSC are rooted in its marked chromosomal instability, a characteristic that contributes to the high diversity of patient-specific genomic complexity, as well as considerable heterogeneity in the tumour microenvironment Schwarz et al. (4); Macintyre et al. (5).

HGSC treatment requires a multimodal approach, involving immediate primary surgery (IPS) or platinumbased neoadjuvant chemotherapy (NACT) followed by delayed primary surgery (DPS) Funingana et al. (6); Kehoe et al. (7). Recent results from phase 3 randomised ICON8 studies suggest that proceeding to DPS after initial NACT is largely determined by the Response Evaluation Criteria In Solid Tumors (RECIST, version 1.1), assessed via Computed Tomography (CT) scans Eisenhauer et al. (8); Bogani et al. (9); Morgan et al. (10). Patients who respond to platinum-based chemotherapy are subsequently treated with poly (adenosine diphosphate-ribose) polymerase or (PARP)-inhibitor, i.e. with (PARPi)-based maintenance therapy Tew et al. (11); Stewart et al. (12); Funingana et al. (13). PARPi, like other DNA damaging agents, demonstrate the capacity to induce immunogenic response and could synergise with ICIs, suggesting the potential to reactivate the immune response in a disease has minimal benefit from ICIs monotherapy. Funingana et al. (13); Strickland et al. (14); Färkkilä et al. (15).

The potential therapeutic synergy between PARPi and ICIs has been tested in the phase II non-randomised MEDIOLA trial Drew et al. (16, 17). The study enrolled gBRCAm platinum-sensitive patients who were candidates for second-line platinum-based chemotherapy. They received 4 weeks of olaparib monotherapy, followed by combination with durvalumab (anti-PD-L1 antibody). This is therefore a very unique cohort consisting of gBRCAm patients treated with PARPi as opposed to SoC platinum-based chemotherapy, with early assessment of response to PARP inhibitor monotherapy before combination with ICIs.

The efficacy of the MEDIOLA combination was measured using RECIST and Gynecological Cancer Intergroup (GCIG) CA-125 criteria Rustin et al. (18), with the primary endpoints being the objective response rate (ORR) and safety (cite Drew 2024 Clin Cancer Research). Despite the utility of these response metrics, they have certain limitations in HGSC. RECIST criteria have been widely utilized as endpoint of early-phase clinical trials, but they are difficult to apply to typical patterns of relapsed disease in HGSC, including multi-site small volume, multi-site lesions, and can fail to accurately capture local patterns of responses or progression either within- or between-metastatic sites Funingana et al. (6). Although tissue-based biomarkers could serve as the gold standard for pharmacodynamic studies, their use is limited by the need for meticulous calibration to the most informative tumour region Qiu et al. (19). This is a significant challenge in HGSC given its inherent multi-site heterogeneity Schwarz et al. (4)Bashashati et al. (20). Additionally, the requirement for invasive biopsy procedures poses logistical and patient comfort and safety concerns Lambin et al. (21). Therefore, while these traditional criteria are useful, they must be used carefully in clinical studies of systemic treatments of HGSC. This emphasizes the importance of developing noninvasive techniques that can accurately capture early response patterns in multi-site diseases like HGSC treated with drug combinations aimed at reactivating the anti-tumour immune response.

Volume and other quantitative imaging measurements, including radiomic features Gillies and Kinahan (22) have the potential to serve as noninvasive biomarkers conveying information about changes in both tumour and microenvironment during therapy, which could be helpful to inform personalised treatments Xue et al. (23). Recent observations suggest that radiomics-based models have the ability to predict response to NACT in ovarian cancer patients, demonstrating the potential role of these features as noninvasive biomarkers Rundo et al. (24).

In this work, different noninvasive imaging-based measurements of spatial and longitudinal heterogeneity were developed and analysed in the MEDIOLA study. The correlation of these measurements of heterogeneity with response assessments based on RECIST were measured at pre-specified time points to identify early markers of response to ICIs-based combination. Special attention was given to comparisons of the local response/progression patterns in different metastatic sites involving peritoneal and non-peritoneal lesions.

## 2 Materials and methods

### 2.1 Patient data

This retrospective study used only pseudo-anonymized data consisting of images and clinical data. Ethics approval by the appropriate Institutional Review Boards and written consent to participate was obtained by the MEDIOLA team, sponsored by AstraZeneca, with trial registered with ClinicalTrials.gov, NCT02734004, and all patients provided written informed consent. More details, including the start of participant recruitment, can be found in a previous publication in Drew et al. (17).

Patients in this trial received olaparib (PARPi) monotherapy for the first four weeks, and then a combination of olaparib and durvalumab (ICI) until disease progression or intolerable toxicity. Imaging was performed with the use of CT or Magnetic Resonance Imaging (MRI) at baseline, four weeks (upon completion of PARPi monotherapy) and every eight weeks thereafter. Response outcomes were assessed using RECIST (version 1.1) Eisenhauer et al. (8) by MEDIOLA clinical investigators. Monthly serum CA-125 concentration values were provided to assess disease response and progression through this marker Rustin et al. (18).

Patients included in this imaging-based subset study (N = 20, identified by alphabetical characters A-T for the remaining of this work) presented germline *BRCA*-mutated, platinum-sensitive relapsed ovarian cancer and their Contrast Enhanced CT (CECT) scans of the thorax, abdomen and pelvis were available at baseline (t_0_), week 4 (t_1_) and week 12 (t_2_, first PARPi-ICI combination assessment). Thirteen (65%) patients carried germline *BRCA1* mutations and seven (35%) had *BRCA2* mutations. All patients had received previous lines of chemotherapy, with nine (45%) patients having received one previous line, four (20%) patients two previous lines and six patients (30%) three or more previous lines. For one patient, the number of previous lines of chemotherapy was unknown.

For the purpose of the analyses in this study, different response assessments were considered. As a result, every patient was classified as responder or non-responder based on the following four categories:

- By RECIST assessment at four weeks: patients are divided into early responders (if partial or complete response was achieved) (ER) or non-responders (for progressive or stable disease) (NR). According to the RECIST 1.1 criteria Eisenhauer et al. (8), complete response is deemed after the disappearance of all the target lesions, with any pathological lymph nodes (target or non-target) having a reduction in short axis to <10 mm. Partial response requires at least a 30% decrease in the sum of diameters of target lesions, taking as reference the baseline sum diameters. These measurements relate to target lesions; when more than one measurable lesion is present at baseline, all lesions up to a maximum of five (and a maximum of two lesions per organ) representing all involved organs are identified as target lesions. Pathological lymph nodes that are measurable and may be identified as target lesions must have a short axis >15 mm.
- By the Best Overall Response (BOR) RECIST assessment over the trial: BOR, as defined by RECIST criteria, represents the best tumour response recorded during the trial. Patients are classified as responders if they achieve partial or complete response at any point, while non-responders are those with only stable or progressive disease throughout.
- Based on the Progression Free Survival (PFS) length: PFS, defined as the time from treatment initiation to disease progression or death, is used to categorise patients. A nine-month cut-off splits the cohort evenly into long responders (LR) with PFS over nine months and short responders (SR) if below.
- Based on the length they stayed in the trial: labelling as long-term enrollees (LTE) those patients whose enrolment time in the study was over one year and as short-term enrollees (STE) otherwise.

### 2.2 Image curation, segmentation and preparation

All lesions identified at baseline (t_0_), week 4 (t_1_) and week 12 (t_2_) CECT scans were manually segmented and cross-validated by two Radiologists in training (VB and DH, both with 6 years’ experience) using the Open Health Imaging Foundation viewer (Open Health Imaging Foundation, Massachusetts Institute of Technology, Cambridge, MA, USA) via its plugin to XNAT, hosted at the local node of the repository established by the CRUK National Cancer Imaging Translational Accelerator (NCITA, https://ncita.org.uk) Doran et al. (25). Lesions were labelled using a numerical code system –within parentheses– upon location:

- Peritoneal sites: Right upper quadrant (RUQ, 2), left upper quadrant (LUQ, 3), mesentery (5), left paracolic gutter (LPG, 6), right paracolic gutter (RPG, 7), pelvis (9), peritoneum other (20), and lesser sac transverse mesocolon (21).
- Lymph nodes (LN): Infrarenal abdominal LN (13), suprarenal abdominal LN (14), supradiaphragmatic LN (15), inguinal LN (17), and other chest LN (16).
- Others: Pleura metastases (11) and lung metastases (19).

Note that the most common sites for ovarian cancer patients (namely omentum and pelvic/ovarian) were only found infrequently (pelvis) or not at all (omentum) in this cohort, as patients had received prior chemotherapy and debulking surgery.

Scans in the original Digital Imaging and Communications in Medicine (DICOM, http://medical.nema.org/) format and segmentation files in the DICOM-RT Law and Liu (26) format were converted to the Neuroimaging Informatics Technology Initiative (NIfTI) format Cox et al. (27) using custom software written in MATLAB (The Mathworks Inc., Natick, MA, USA) version R2019b for subsequent feature extraction.

### 2.3 Metastatic sites and anatomical distribution

Anatomical networks of the metastases were constructed to represent the distribution of the disease. Each node in the network corresponds to a metastatic site (See **Supplementary Figure S1**) and they are interconnected for every patient and time point. Information from multiple patients was combined into a single network by proportionally scaling the size of the nodes and the thickness of the edges according to the number of patients that were found with those.

The number of edges (patients with disease in the sites on both ends) was taken as a measure of the complexity of the networks. Additionally, anatomical dissemination was also quantified by the total number of lesions (individual fully-connected volumes within each site) and two distance measurements: total intersite distance (i.e. the sum of all Euclidean distances between site centres of mass in the image space) and the maximum distance between two sites Cottereau et al. (28).

Complementarily, co-occurrence matrices were created to illustrate the concomitant presence of sites within patients and were of use to identify pairs of sites with higher likelihood to appear at the same time.

### 2.4 Volume and radiomic features extraction

Three first-order radiomic features that quantify heterogeneity (namely energy, entropy and uniformity) for every site, patient and time point were extracted using the open-source Python package PyRadiomics version 3.0.1 Van Griethuysen et al. (29). Higher order radiomic features were not taken into account given the small sample size and the less obvious interpretability of such features.

The pre-processing included re-sampling the images to the average voxel size over the range of values of the cohort (0.801×0.801×3.764) mm using Welch sinc interpolation method to minimise effects of different reconstructed voxel sizes Escudero Sanchez et al. (30).

### 2.5 Volumetric response over time

The volume of each site for every patient and time point was used to evaluate volumetric treatment response. The effect of the different treatment arms to the total volume was compared by extracting the relative change in volumes between t_0_ and t_1_ (first arm, four weeks of PARPi only) and t_1_ and t_2_ (second arm, eight weeks of PARPi + ICI). The peritoneal and non-peritoneal sites (see list of sites per region above) contribution to total volumetric burden –i.e. the sum of volumes for all sites– was also calculated.

### 2.6 Radiomics analyses

For every patient, the values of the radiomic features Energy, Entropy and Uniformity were extracted for all sites independently, and the median was used to quantify their overall value. Additionally, as an alternative measurement of the spatial heterogeneity presented by these variables across different sites, their range values were also calculated. Moreover, a summed value of each feature was obtained by adding up its value at every site. When the whole cohort was analysed over time, one patient (patient A) was excluded as no lesions were identified at t_2_ and the Wilcoxon signed-rank tests on paired samples cannot be performed under the absence of measures.

### 2.7 Statistical analysis

Descriptive and inferential statistics were applied to imaging-derived features. Nonparametric tests were conducted and considered statistically significant if *p ≤*0.05. For paired samples (i.e. same patient, different time point or same patient, different lesion site), Wilcoxon signed-rank tests on paired samples were used. A Kruskal-Wallis test was performed to assess differences between defined groups. All statistical analyses were conducted using R (31) version 4.2.1.

## 3 Results

### 3.1 Patient stratification

**Figure 1:**
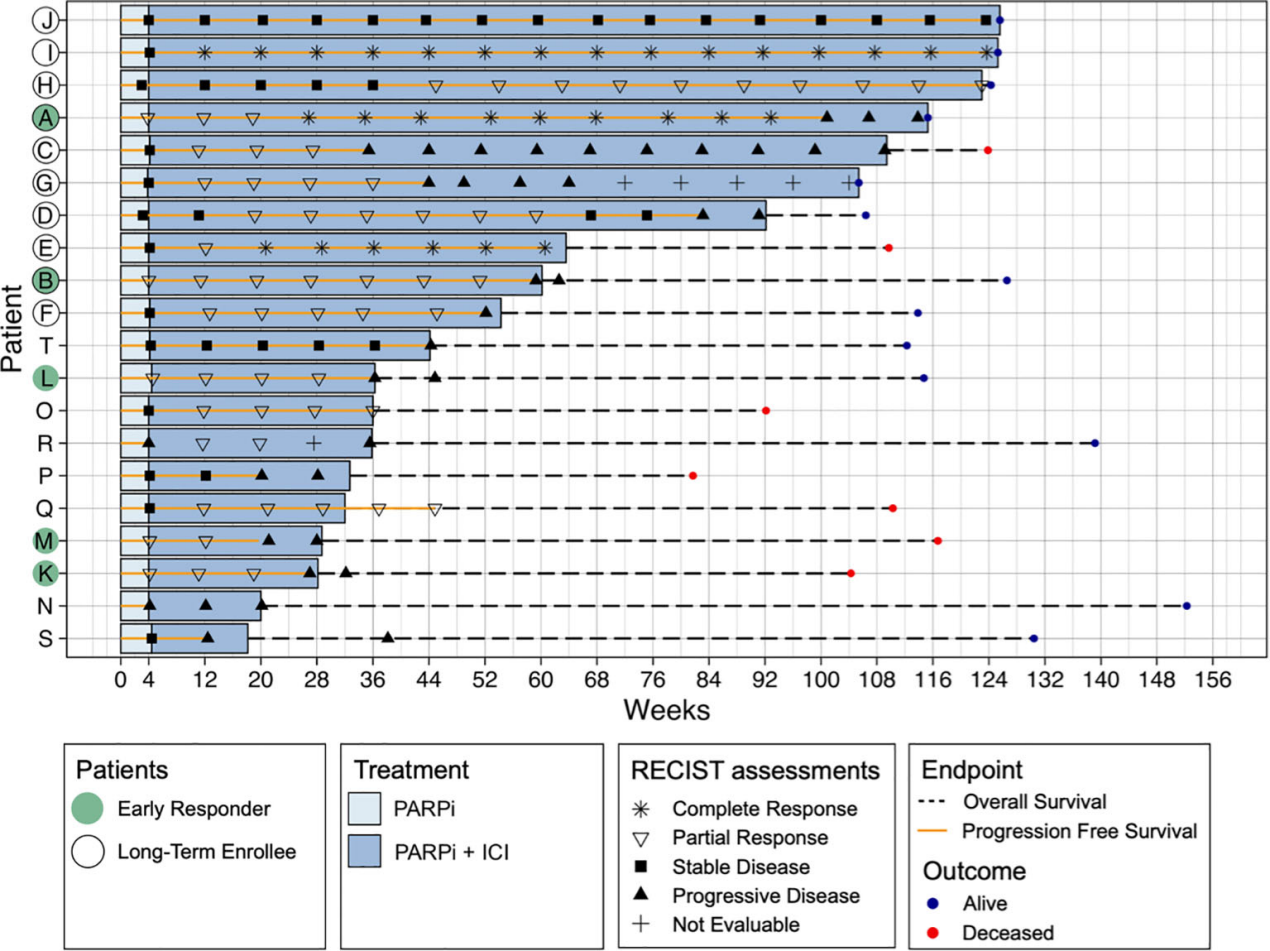
Swimmer plot of all patients (N = 20) showing all RECIST assessments, Progression Free Survival (PFS), Overall Survival (OS) and outcome PFS represents the time from treatment initiation to disease progression or death, while OS measures the time from treatment initiation to death from any cause. Patients are categorised based on RECIST response at four weeks (early responders, ER) and Best Overall Response (BOR), which is based on RECIST assessments throughout the entire study, classifying patients as responders (complete or partial response) or non-responders (stable or progressive disease). Additionally, patients are grouped by PFS length, with a cut-off at nine months, where those exceeding this threshold are considered long-term enrollees (LTE). Treatment type (PARPi or PARPi + ICB) is also indicated.

Patients were enrolled in the clinical trial for an average of 64 weeks (range 18–126 weeks, median 49 weeks). Separated by a cut-off at one year, ten patients were short-term enrollees (STE) and ten were long-term enrollees (LTE) (**Figure 1**). The average Progression Free Survival (PFS) time was 53 weeks (range 4-124, median 44 weeks), with 9 patients below nine months (short responders, SR) and 11 above (long responders, LR). According to their RECIST assessments, five patients were early responders (ER) and 15 non-responders (NR) at four weeks, and 14 achieved partial or complete response over the entire trial versus 6 who did not, following the Best Overall Response (BOR) RECIST assessment criteria.

None of the patients had progressed by CA-125 assessment within the first 12 weeks, hence CA-125 was not used as a response measurement in further analyses.

### 3.2 Comparison of response assessment measurements

**Figure 2:**
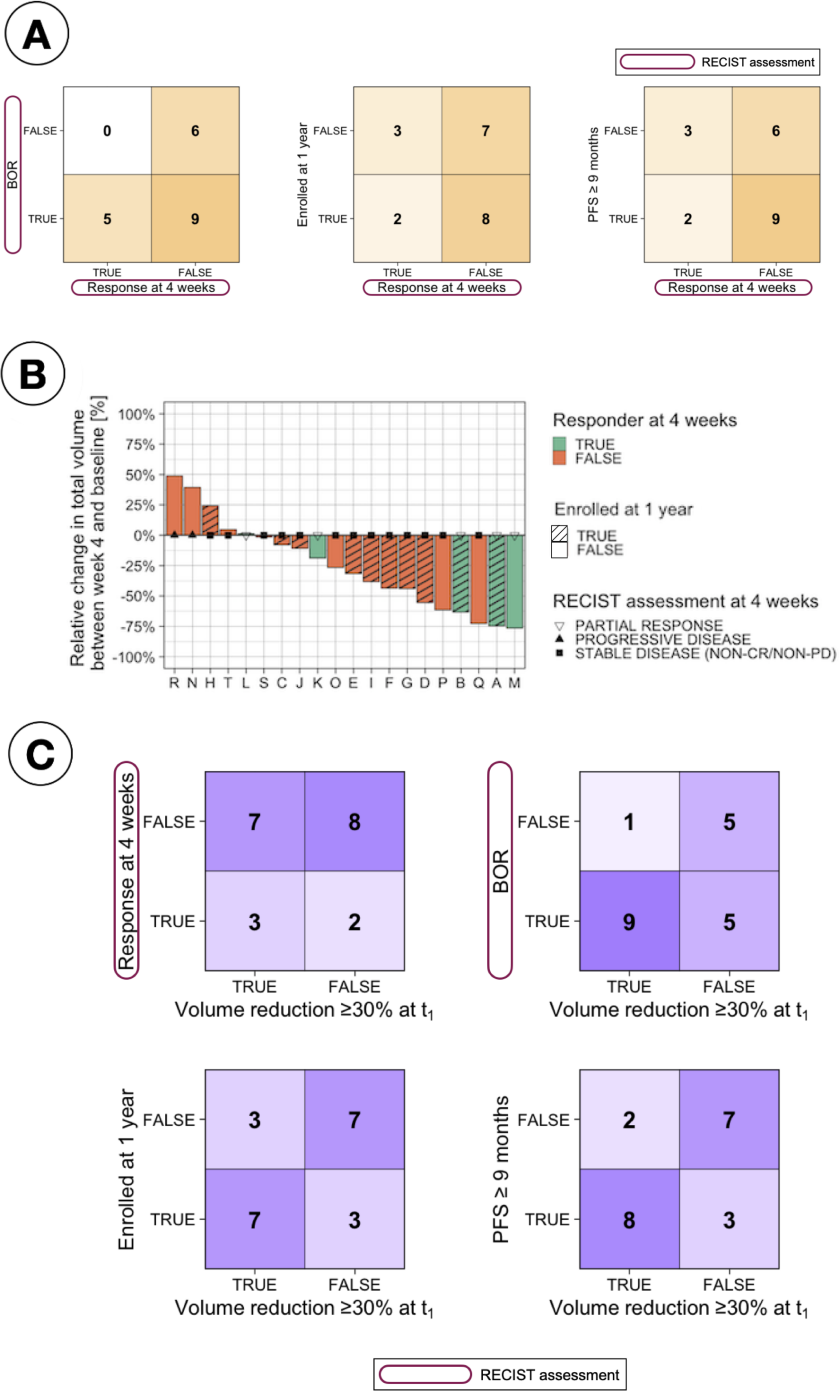
Comparison of response assessments. **(A)** Confusion matrices comparing RECIST response at 4 weeks with different patient stratifications, including Best Overall Response (BOR, defined as the best assessment recorded throughout the study based on RECIST criteria), long-term enrollee status (LTE, patients enrolled for ≥1 year), and progression-free survival (PFS) over nine months. **(B)** Waterfall plot showing relative change in total tumour volume between baseline and 4 weeks, stratified by RECIST response at 4 weeks and long-term enrollee status (LTE vs. STE, where STE refers to short-term enrollees with less than one year on study). **(C)** Confusion matrices evaluating the relationship between tumour volume reduction (≥30% at t_1_) and various response criteria, including RECIST response at 4 weeks, BOR, LTE status, and PFS ≥9 months.

**Figure 2** compares the different response assessment measurements analysed in this study. A comparison with the earliest measurement available in the trial, which is RECIST response at four weeks, is presented in **Figure 2A**. The confusion matrices clearly show that the early assessment obtained with RECIST is not a good indicator of long-term patient response to treatment. In particular, it was found that many patients who did not respond according to RECIST at 4 weeks (15 patients) did achieve response during the overall treatment (BOR, 9 patients - 60%), were long time enrollees in the trial (LTE, 8 patients - 53%) or had at least 9 months of PFS (LR, 9 patients - 60%).

The relative change in total volume between t_0_ and t_1_ was also studied as an early assessment of response, motivated by the results observed in **Figure 2B**, which indicate that several patients deemed as NR at 4 weeks based on RECIST measurements presented a large change in total volume. Establishing a cut-off at 30% in volumetric changes [based on the same relative change of 30% used in 2D by RECIST Eisenhauer et al. (8)] to classify patients as responders or non-responders, a better correlation between this early response measurement and long-term response was found, as observed in **Figure 2C**.

### 3.3 Relapsed ovarian cancer patients characterised by high spatial heterogeneity

**Figure 3:**
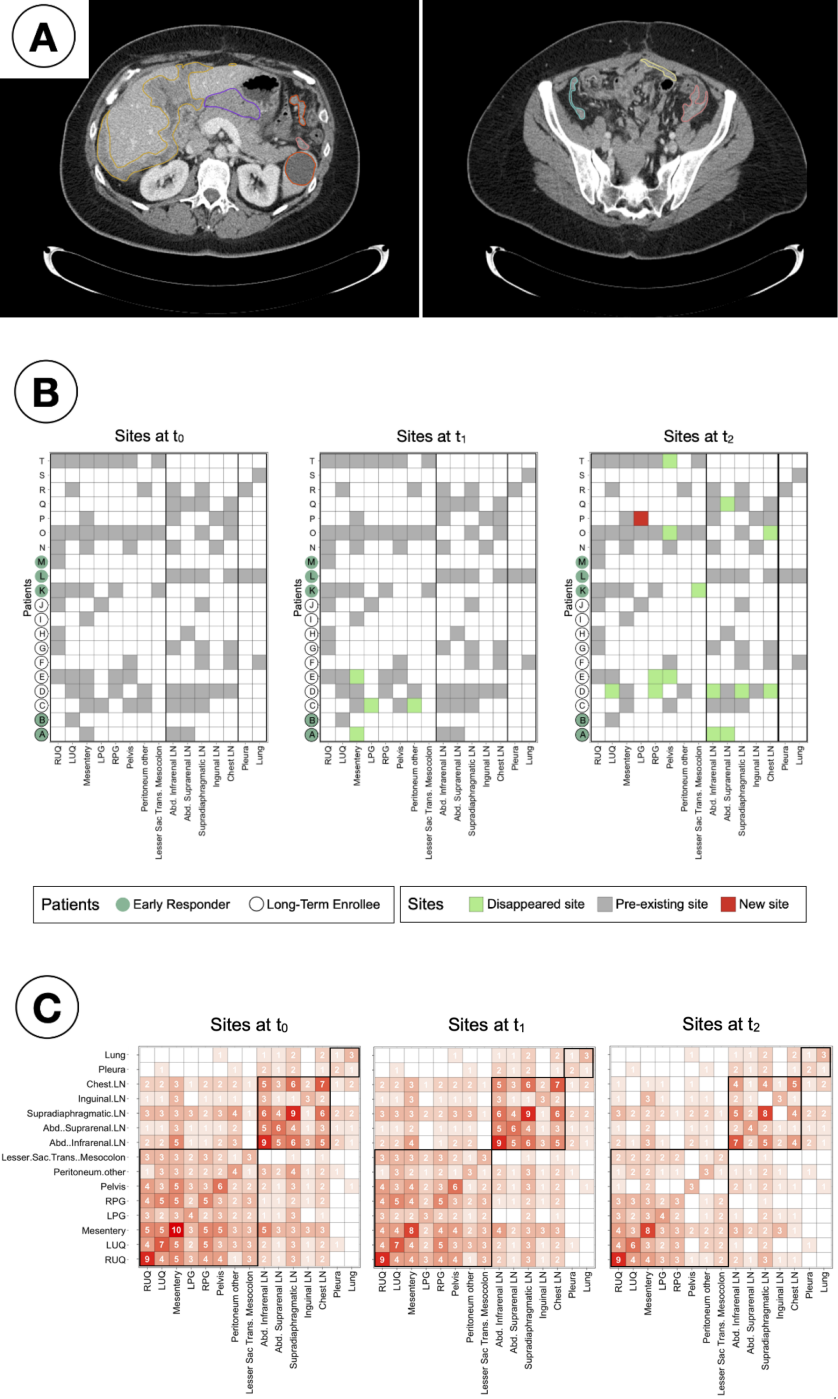
Study of inter-patient metastatic sites distribution. **(A)** Example 2D CT slices of a single patient and time point, showing segmented lesions in the peritoneal corresponding to: Right upper quadrant (yellow, left); Left upper quadrant (orange, left); Mesentery (light yellow, right); Left paracolic gutter (salmon, both); Right paracolic gutter (cyan, right); Lesser sac transverse mesocolon (purple, left). **(B)** Checkers of the sites found in every patient and time point. **(C)** Co-occurrence matrices showing the number of patients for simultaneous sites at every time point. The sites are in framed in black by region (peritoneal, lymph nodes and other).

Lesions were identified and segmented in 15 distinct anatomical sites across the cohort (**Figure 3**). All patients had metastatic disease at enrolment, with a median of four metastatic sites (range 1-10) per patient at all time-points; being the mesentery the site with the highest occurrence, present in ten (50%) patients at baseline. Non-significant differences were found (Kruskal-Wallis testing, *p >* 0.05) when comparing, for any of the response assessment classifications, i) the median total number of sites or ii) the median number of peritoneal sites, lymph nodes or other sites at any time point (**Supplementary Table S1**). As observed in **Figure 3B**, after the first treatment arm (PARPi only) disease in some peritoneal sites only disappeared: the mesentery disappeared for two patients and, for a third patient, lesions in both LPG and peritoneum (other) disappeared. Additional sites, including several LN disappeared for 7 patients over the second arm treatment (PARPi + ICI), whilst only in one patient a new site (LPG) appeared.

Co-occurrence matrices (**Figure 3C**) were created to identify clusters of sites that had the tendency to appear together. It was observed that over 75% of the patients (85% at t_0_ and t_1_, and 75% at t_2_) that presented lesions in any of the lymph nodes were found to have lesions in other LN too.

### 3.4 Anatomical networks to assess spatial heterogeneity

**Figure 4:**
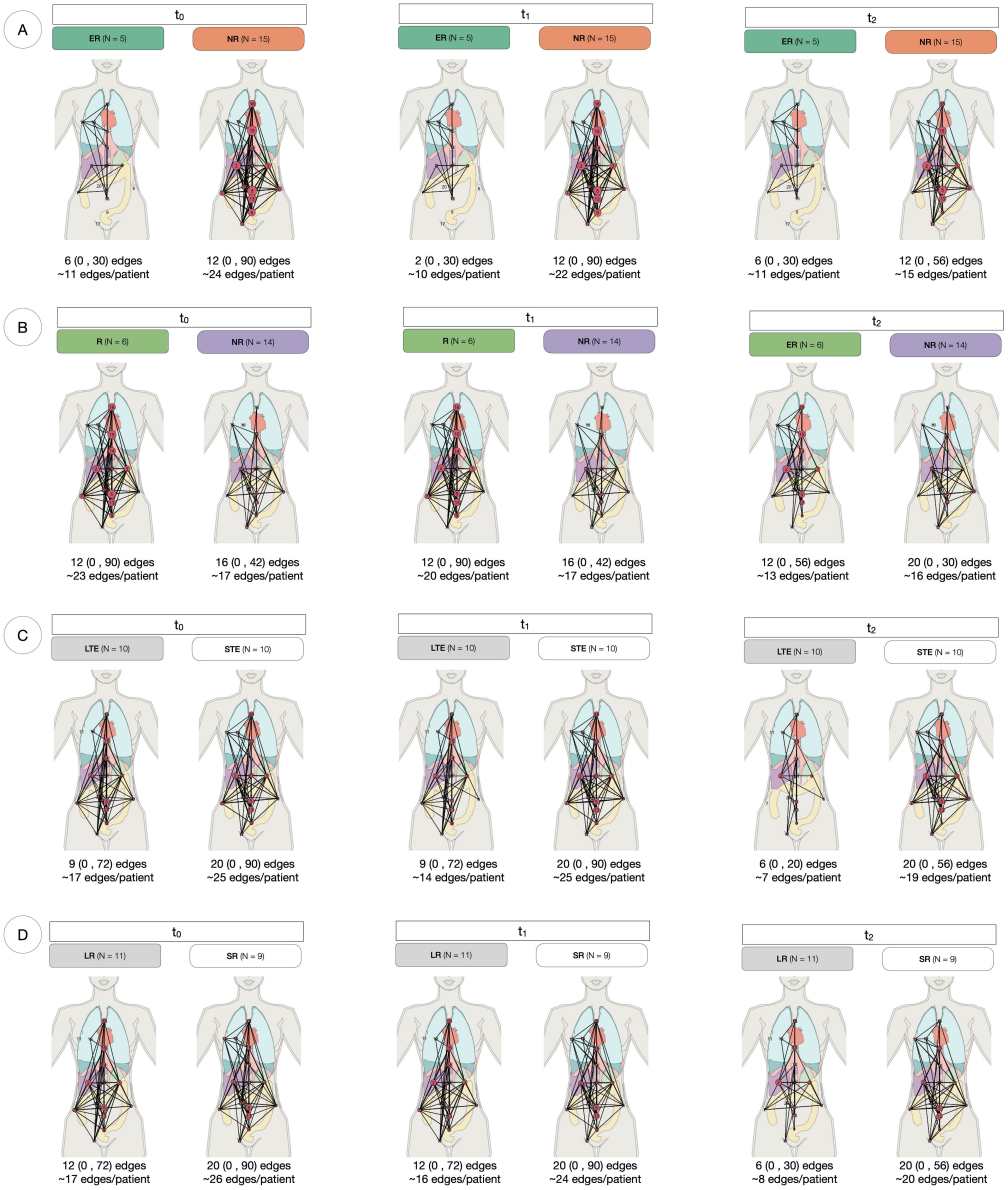
Anatomical networks for every response assessment. Each panel represents the anatomical connectivity of metastatic sites, where nodes correspond to lesion locations and edges indicate anatomical connections between them. The median, range (in parentheses), and mean number of edges per patient are provided for each group at t_0_, t_1_ and t_2_. **(A)** At RECIST assessment at 4 weeks, Early Responders (ER) tend to have fewer edges than Non-Responders (NR) at all time points. In NR, the median number of edges remains constant over time. **(B)** For Best Overall Response (BOR), responders (R) maintain a stable median connectivity, while NR exhibit increasing connectivity over time. **(C)** Long-term enrollees (LTE) show a consistent median number of edges across timepoints, whereas Short-term enrollees (STE) experience a decline in connectivity during the second treatment arm (t_1_ to t_2_). **(D)** Long Responders (LR, PFS ≥ 9 months) have lower connectivity than Short Responders (SR, PFS *<* 9 months) at all time points, with a 50% decline in median edges at *t*_2_.

**Figure 5:**
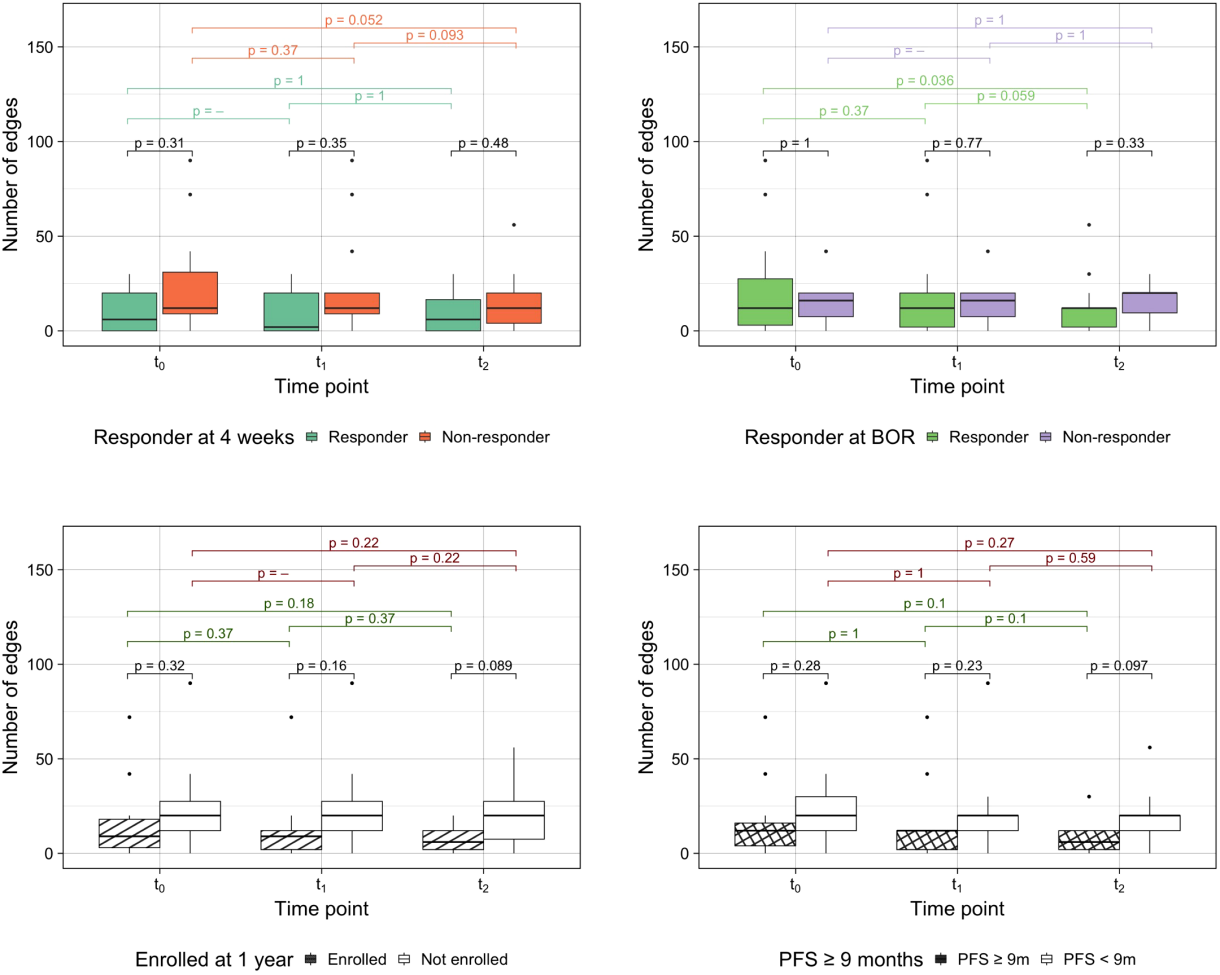
Number of edges by response assessment measurements. Differences between groups were assessed performing Kruskal-Wallis testing and intra-group longitudinal comparisons were tested with Wilcoxon signed-rank tests.

As a novel approach to measure spatial heterogeneity and changes thereof, anatomical networks were constructed for each patient and time point (**Figure 4**). The number of edges of every patient can be found in **Supplementary Figure S2**. While the median number of edges at every time point remained constant at 12 edges, the mean decreased (21 at t_0_, 19 at t_1_ and 14 at t_2_). The minimum value of the range was 0 for the three time points (given that four patients in the cohort had one site only), but the maximum decreased from 90 at t_0_ and t_1_ to 56 at t_2_. **Figure 5** compares the number of edges for every time point and response measurement assessment. The median number of edges is lower for the responders at all time points for every response assessment measurements; however, the differences are not significant (Kruskal-Wallis testing, *p >* 0.05). Moreover, when assessing longitudinal changes in the number of edges for each classification, only BOR responders present a significant decrease in the median number of edges at t_2_ when compared to baseline (paired samples Wilcoxon signed-rank tests, *p <* 0.05). Nevertheless, the following trends were observed in the average number of edges per patient (**Figure 4**): there is typically a decrease in all categories, smaller during the first treatment arm, but more noticeable in the second arm, especially for responders in the long term measurements (LTE and ≥9 months PFS) for which the value halves.

### 3.5 Dissemination measurement of spatial heterogeneity over time

**Figure 6:**
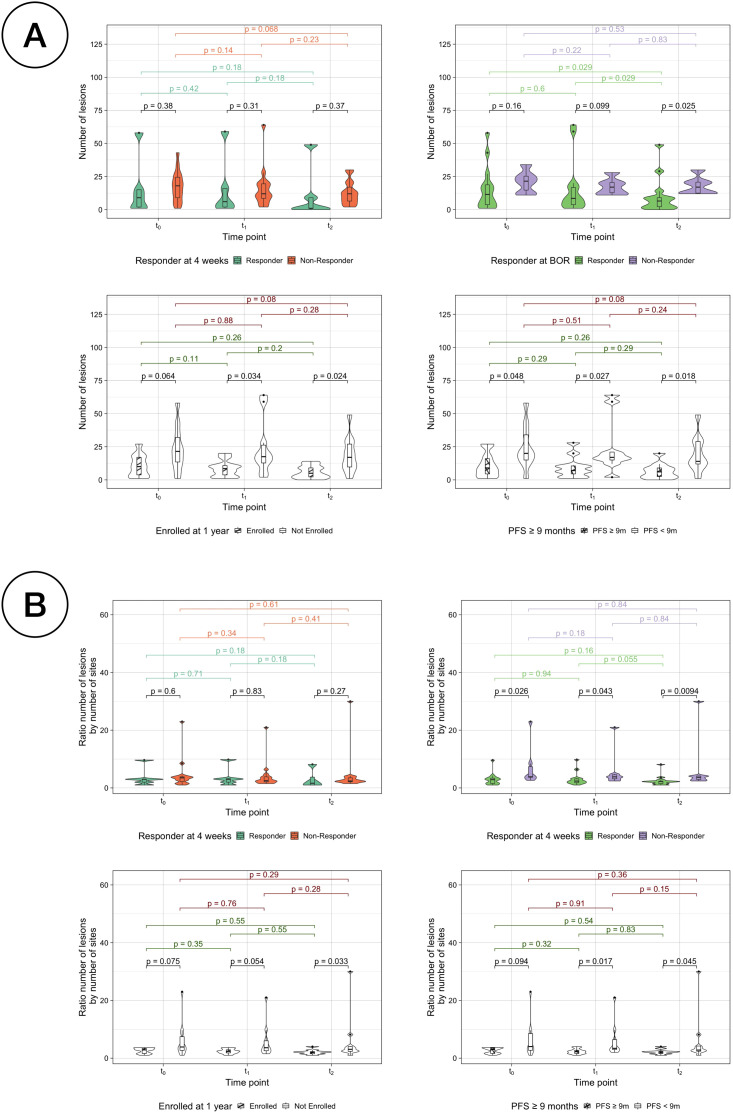
Comparison of number of lesions per classification. **(A)** Number of lesions by response assessment measurements for responders vs non-responders. **(B)** Ratio between the number of lesions by the number of sites by response assessment measurements for responders vs non-responders.

Firstly, anatomical dissemination was quantified by the total number of individual volumes or lesions (**Supplementary Table S2**). The median number of lesions for the whole cohort at baseline was 14.5 (range 1-58), 11.5 lesions (1-64) at week 4 and 9 lesions (0-49) at week 12. The number of lesions significantly decreased for the whole cohort from t_0_ to t_2_ (paired samples Wilcoxon signed-rank tests, *p* = 0.02) (**Supplementary Figure S6**). Kruskal-Wallis testing was used to evaluate the differences between the different response assessments. For the RECIST assessments, the number of lesions was significantly different only in BOR responders vs non-responders at t_2_ (*p* =0.02). In case of PFS assessments, the number of lesions between SR and LR were significantly different at every time point (*p* =0.05, *p* =0.03 and *p* =0.01, respectively) and for LTE vs STE the differences were significant at t_1_ and t_2_ (*p* =0.03 and *p* =0.01, respectively) (**Figure 6A**). To reduce the effect of the number of sites to the number of lesions (Pearson correlation coefficient *r* =0.7) (**Supplementary Figure S7**), the ratio of them was also analysed and resulted to be significantly different for LTE vs STE at t_2_ (Kruskal-Wallis testing, *p* =0.03), for PFS LR vs SR at t_1_ and t_2_ (*p* =0.02 and *p* =0.04) and for BOR responders vs non-responders at every time-point (*p* =0.03, *p* =0.04 and *p* =0.01) (**Figure 6B**).

In addition, the sum of intersite distance and the maximum distance between two sites were studied. For the whole cohort of patients, no significant differences were observed between any time points except for the maximum distance, the median of which was significantly different between t_0_ and t_1_ (paired samples Wilcoxon signed-rank test, *p* =0.03) (**Supplementary Figure S3**). None of the dissemination distance measurements were significantly different when comparing groups ER vs NR, R vs NR by BOR, LTE vs STE or SR vs LR by PFS (Kruskal-Wallis testing, *p >* 0.05) (**Supplementary Figures S4**, **S5**).

### 3.6 Volumetric changes over time as assessment of treatment response

**Figure 7:**
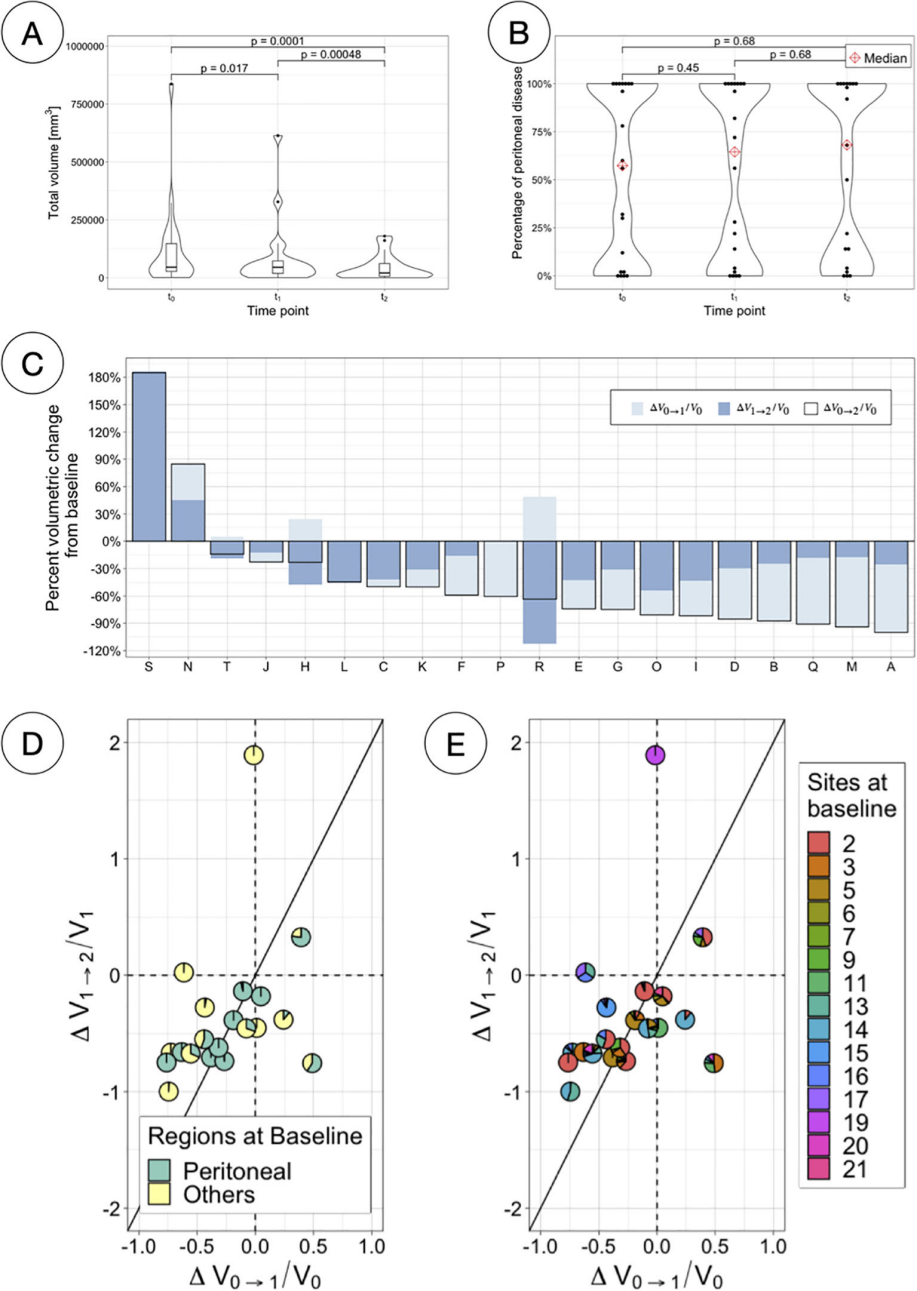
Total volumetric responses. **(A)** Total volumetric burden at each time-point. **(B)** Percentage of total volume corresponding to peritoneal disease at each time-point. **(C)** Percentage volumetric change from baseline per patient, indicating the changes for different time ranges (first and second treatment arm independently and combined). **(D)** Scatterpies plot comparing relative changes in total volume between the two therapy arms, separating components due to peritoneal and other disease. **(E)** Scatterpies plot comparing relative changes in total volume between the two therapy arms, separating components due to each disease site.

Total volumetric burden decreased significantly for all patients as treatment advanced (paired samples Wilcoxon signed-rank tests, *p <* 0.05) (**Figure 7A**, **Supplementary Figures S8**, **S9**); however, the percentage of disease confined in the peritoneum did not significantly differ at any of the time points (paired samples Wilcoxon signed-rank tests, *p >* 0.05) (**Figure 7B**). Most patients total volumetric burden (13 patients, 65%) decreased both over the first four weeks of PARPi monotherapy (median -29%, range -76%–+48% from t_0_) and over the following eight weeks of combined therapy (median -28%, range -112%–+186% from t_1_) (**Figure 7C**). Only two patients (P and S) presented a decrease in volume during the first treatment arm followed by an increase in volume in the second arm. For many patients instead (N = 14), the volume decreased over both treatment arms and therefore appear in the third quadrant when comparing the change in volume between the two therapy arms (**Figures 7D, E**). Moreover, although for 13 of these patients the major contribution to the total volume decrease over the 12 weeks happened between t_1_ and t_2_, two important caveats are to be taken into account: i) that initial volumes were significantly lower at t_1_ (see **Figure 7A**) and ii) that patients were in combined therapy for double the amount of time. To account for such differences in **Figures 7D, E**, the changes in volume presented are relative to the initial volume in that range, and the diagonal (around which most of the cases appear), is drawn to compensate for the double amount of time in the second arm.

**Figure 8:**
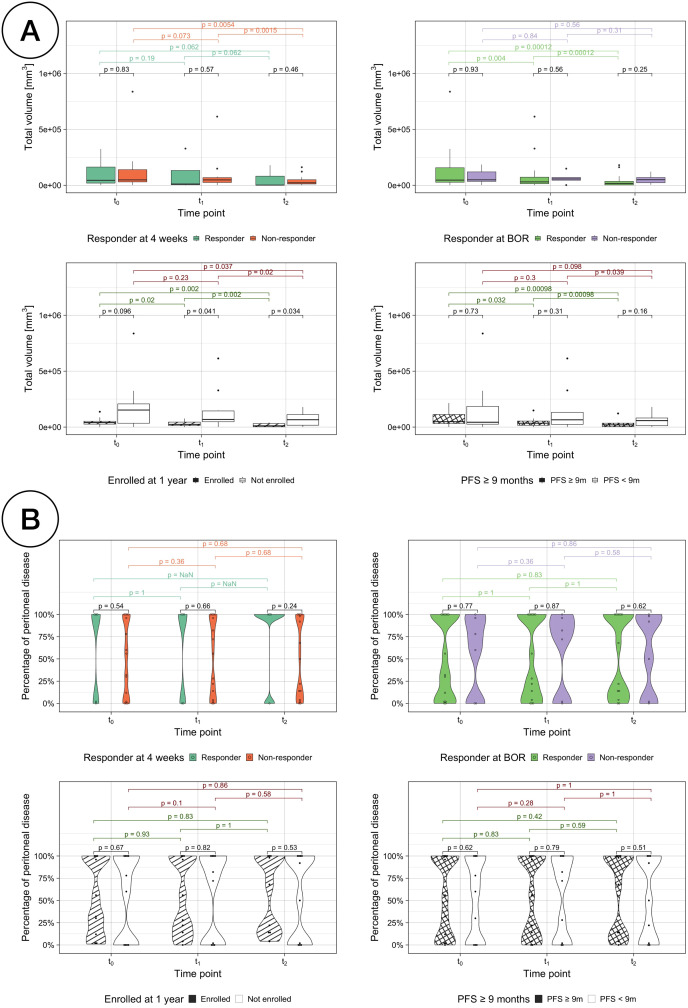
**(A)** Total volumetric burden by treatment response assessments. Comparison of the total volumetric burden for responders vs non-responders with each different treatment response assessment: RECIST response at 4 weeks (top left), BOR (top right), enrollment length (bottom left) and PFS (bottom right). **(B)** Percentage of disease volumetric burden in the peritoneal cavity by treatment response assessments.

Volumetric changes over time were also assessed against each traditional treatment response assessment (**Figure 8A**). While the median volume is lower in the responder group for every comparison assessment and time point, the difference is only significant between classes when assessing LTE vs STE (Kruskal-Wallis testing, *p <* 0.05). Responders by enrollment time, PFS and BOR show a significant decrease over time (paired sample Wilcoxon signed-rank tests, *p <* 0.05).

The volumetric distribution inside and outside the peritoneal cavity was also studied for all the response assessment classifications and no statistically changes were found neither between classes nor over time (**Figure 8B**). However, for responders according to RECIST assessment at 4 weeks, their disease was either exclusively located outside or inside the peritoneal cavity, with the exception of one patient with a relative volumetric burden in the peritoneal cavity of just 1.05%.

### 3.7 Additional radiomics-based biomarkers to assess response

**Figure 9:**
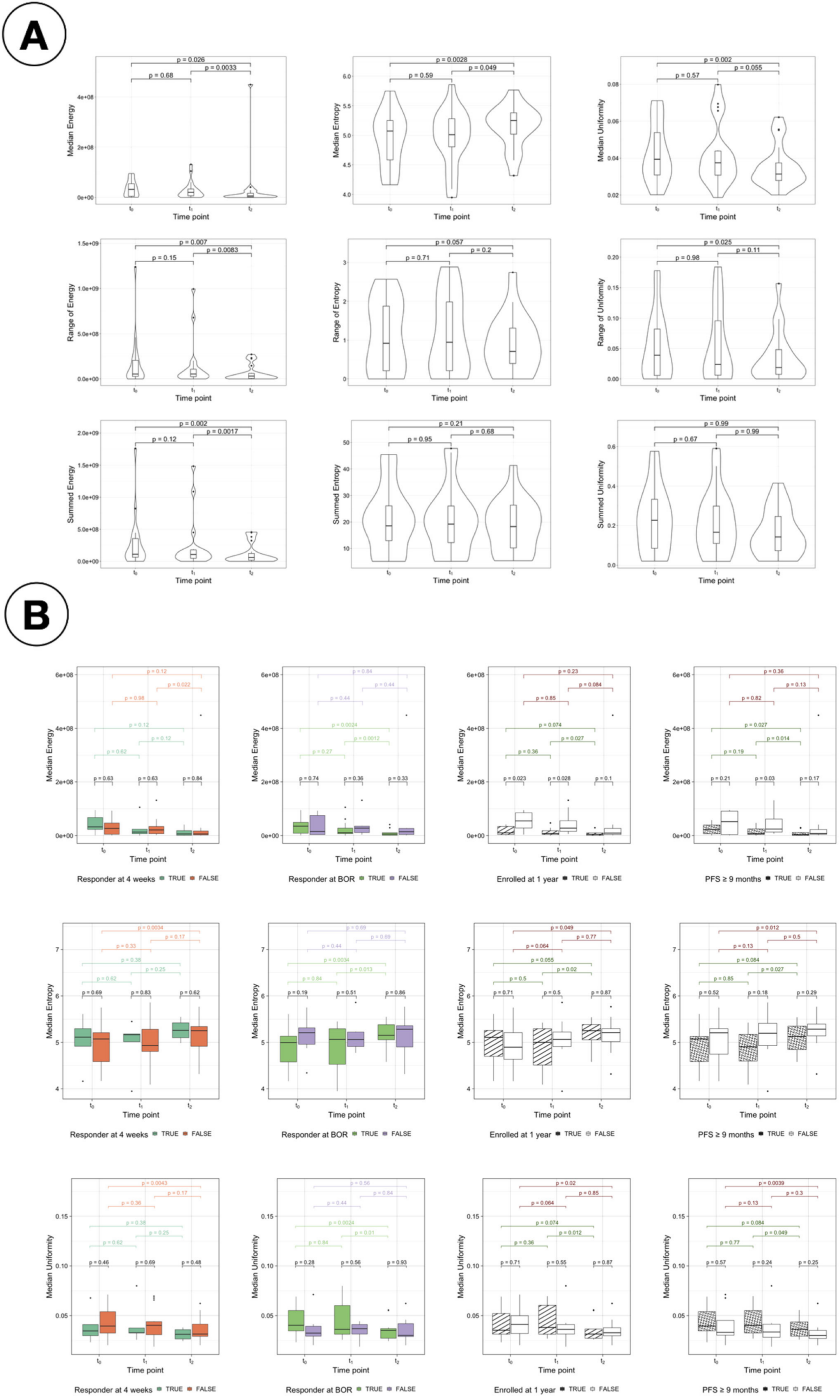
Study of radiomics features. **(A)** Change in radiomics features for intrapatient measurements of median, range and sum. Change in median (top), range (middle) and summed (bottom) values of the radiomics features Energy (first column), Entropy (second column) and Uniformity (third column) for all patients. **(B)** Comparison of the median values for energy, entropy and uniformity for the response assessments. Comparison of the median values of the radiomic features Energy (top), Entropy (middle) and Uniformity (bottom) for responders vs non-responders with each different treatment response assessment: RECIST response at 4 weeks (first column), BOR (second column), enrollment length (third column) and PFS (forth column).

Median, summed and range values of the first-order radiomic features Energy, Entropy and Uniformity for all patients were analysed and presented in **Figure 9A**. Regarding Energy, changes observed in the second treatment arm and over the twelve weeks of therapy analysed in this study (from t_0_ to t_2_) were significant, but they were not for the first arm. Changes were less pronounced, and therefore significant, for the other two features. In the case of Entropy, the only significant changes observed correspond to the median value during the second arm and from t_0_ to t_2_. Similarly, changes were significant for Uniformity only during the second arm for its median value, and from t_0_ to t_2_ for median and range.

**Figure 9B** presents the comparison of the median values of Energy, Entropy and Uniformity for responders vs non-responders for the four response assessment measurements considered (see **Supplementary Figure S10** for the summed values and **Supplementary Figure S11** for the comparison of the range values). Significant differences were found at t_0_ and t_1_ for LTE vs STE and for PFS responders vs non-responders at t_1_ using median Energy. Differences between responders and non-responders are also observed in the distribution of values of Entropy and Uniformity; however, they were not significant.

## 4 Discussion

In this analysis, the use of distribution studies over individual-site analyses was motivated by the small size of the cohort (N = 20 patients) together with the heterogeneous spread of the disease into 15 different sites, with no common site appearing in all patients. The use of checkers (**Figure 3B**) allowed for the visualisation of individual and regional (peritoneum, lymph nodes and other sites) tendencies to treatment response. Through them it was possible to establish that the desired complete response (i.e. disappearance) was found in the study subset within the first four weeks of PARPi monotherapy only for some peritoneal sites, and additionally in lymph nodes for some patients after the eight weeks of PARPi + ICI, highlighting the potential benefit of the combined treatment.

Distribution studies included the consideration of the total number of sites, the modelling of anatomical networks and the building of co-occurrence matrices. The median number of sites was non-significantly different to distinguish between responders and non-responders at any time point. However, it was found that the number of lesions could be used to differentiate between long-term responders and non-responders, but at baseline it was only statistically significant for PFS (**Figure 6A**). The average number of edges per patient was found to generally decrease as the treatment advanced and differences were observed between comparison groups, especially in assessments of long-term response (**Figures 4**, **5**). The rationale behind using anatomical networks is to visualise disease heterogeneity, providing a deeper understanding of how lesions are interconnected across the body. While the current sample size limits the immediate predictive utility of this approach, increasing the number of patients is likely to enhance its significance and robustness. Moreover, combining anatomical networks with additional sources of information, such as changes in volume or radiomic features, could further refine our understanding of disease progression. Radiomic textural features, for instance, could help revealling patterns of clonal expansion, offering insight into how different metastatic sites interconnect. This integrated approach holds promise for more personalised patient management by identifying regions of clonal spread, informing treatment targeting, and improving longterm monitoring, particularly in differentiating responders from non-responders. Finally, co-occurrence matrices proved that the cluster of lesions in the lymph nodes was the most highly correlated, implying that the presence of lesions in one of them in over 75% of the cases translated with the existence of other lesions in other LN.

In addition, changes in volume were studied as a measurement to assess response. Firstly, it was found that changes in volume between t_0_ and t_1_ (after the 4 weeks of PARPi monotherapy) could potentially be used as an early indicator of response to ICIs-based combination, in fact better correlated to long-term response than the current early assessment done using RECIST at 4 weeks as shown in **Figure 2**. This demonstrates the advantage of considering 3D changes in the whole disease burden as opposed to only 2D changes in selected lesions in cancers as complex as HGSC. Indeed a reduction in total tumor volume is an intuitive measure of therapeutic efficacy and multiple studies have demonstrated that volumetric measurements can be more sensitive to subtle tumor responses than unidimensional metrics, potentially offering earlier detection of treatment efficacy or resistance. Furthermore, assessing tumor burden across multiple anatomical sites (i.e., spatial heterogeneity) provides additional insights into metastatic potential and tumor biology as reduction in inter-lesion variation often correlate with better outcomes or improved control of disseminated disease Aerts (32). The findings of the present work align with this body of evidence, showing that changes in both total volume and spatial heterogeneity offer meaningful markers of response in HGSC. It is important to highlight that, although RECIST remains a widely accepted standard, it captures only up to five lesions and focuses on unidimensional changes, and this can underestimate the overall burden of disease and may not detect early volumetric reductions or evolving patterns of spatial heterogeneity.

Total volumetric burden was found to decrease significantly as treatment advanced (**Figure 7A**) but the distribution of the disease in regions (inside vs outside the peritoneal cavity) remained constant (**Figure 7B**). Scatterpies (**Figures 7D, E**) have been introduced in this work to compare the relative change in volume over the first four weeks under monotherapy to the following eight weeks of combined therapy, while illustrating the regional and sites distribution at baseline. More sites were found to respond better during the combination therapy period.

Other first-order radiomic features characterising heterogeneity were analysed. Significant changes were found in median values of Energy, Entropy and Uniformity for all patients during the second treatment arm, and only in Energy when comparing long term and short term enrollees at t_0_ and t_1_ (baseline and after the first treatment arm) and for PFS at t_1_. These results remain to be further confirmed with larger datasets, and the intrinsic dependencies of these features with volume need to be corrected Escudero Sanchez et al. (30) Shafiq-Ul-Hassan et al. (33) Shafiq-ul Hassan et al. (34), but they show a preliminary indication that such noninvasive biomarkers might be useful in stratifying patients according to their response to treatment in the future.

To our knowledge, other studies evaluating combined PARPi and immunotherapy (PARPi-ICI) in a sequential approach are still ongoing. Indeed, most PARPi–ICI trials have used concurrent therapy, which has produced mixed results depending on patient selection. Examples of such trials with results using concurrently PARPi–ICI are the following:

- TOPACIO/KEYNOTE-162 (NCT02657889) for Ovarian (platinum-resistant) and Triple-Negative Breast Cancer (TNBC). Niraparib + Pembrolizumab. Phase I/II (completed) Konstantinopoulos et al. (35)Vinayak et al. (36).
- MEDIOLA (NCT02734004) – Multi-cohort basket: gBRCA-mutated Ovarian Breast; also Gastric and Small Cell Lung Cancer (SCLC). Olaparib + Durvalumab. Phase II (completed initial cohorts for sub-studies on breast, gastric cancer and SCLC) Domchek et al. (37)Krebs et al. (38).
- JAVELIN PARP Medley (NCT03330405) – Basket trial (multiple solid tumors and molecular subsets). Talazoparib + Avelumab (PARP inhibitor + anti–PD-L1). Phase I/II (completed) Yap et al. (39).
- CheckMate 9KD (NCT03338790, Cohort A) – Metastatic Castration-Resistant Prostate Cancer (mCRPC). Rucaparib + Nivolumab. Phase II multi-arm trial (one arm with PARPi–ICI) (completed) Fizazi et al. (40).
- KEYNOTE-365 Cohort A (NCT02861573) – mCRPC post-docetaxel. Olaparib + Pembrolizumab. Phase Ib/II (completed) Yu et al. (41).

These works are small or earlier-phase studies that investigated the safety and preliminary efficacy of such combinations. Sequential approaches (such as induction PARP inhibitor followed by PARP inhibitor and immunotherapy treatment) are still under evaluation, and no conclusive success has been observed yet. In this paper, we demonstrated how our imaging-based methodology enhances the assessment of tumor dynamics, potentially guiding the integration of these combination regimens into clinical practice.

The main limitations of the analyses presented in this paper were twofold. Firstly, the small size of the patient cohort limited the studies that could be done and impacted the statistical significance of the tests performed. Trends and differences were observed across the different studies in this paper, however statistical tests show no significance in many cases with the small dataset used. In the future, these analyses should be further confirmed, and expanded including higher-order radiomic features as well, with a larger cohort of patients.

Secondly, although the comparison of response and changes between the two treatment arms (PARPi monotherapy vs PARPi + ICIs) was one of the aims of this study, the results are to be interpreted in the light of two caveats: i) that the time between measurements (t_0_ and t_1_ vs t_1_ and t_2_) was double (8 weeks) for PARPi-ICI than for PARPi alone (4 weeks); and ii) that the baseline volume for the combined therapy had reduced during the first 4 weeks of treatment for most patients (see **Figure 2B**), hence the initial conditions for both treatment arms were different.

Future work should address these limitations with larger datasets. At the moment, however, studies evaluating combined PARPi and immunotherapy with a sequential approach are limited or still ongoing. The imaging-based assessments of response developed in this work could therefore be tested further in the near future in the context of other trials that aim to compare arms with different treatments or combinations thereof. Also, obtaining a full segmentation of all sites for these patients is challenging, time-consuming, and impractical to be done manually; hence fully automated solutions for the segmentation of ovarian cancer Buddenkotte et al. (42), as well as other metastatic cancers, will be of the upmost importance in the near future. Indeed, creating an automated pipeline for segmentation and feature extraction would also help reduce the variability associated with manual segmentations, similar to the intra- and inter-reader variability that can be observed in other manual assessments such as RECIST.

Future studies should also consider more long-term patient data when available in order to expand the longitudinal assessment capabilities of this method for long-term outcomes. Other confounding factors that could impact the results should be also further investigated in the future, such as patient demographics, prior therapies, etc. Finally, beyond the purpose of this analysis, other emerging assessments alternative to RECIST could be explored and compared to as well in future studies.

Despite the limitations mmentioned, the imaging-based methodology proposed in this work enhances the assessment of tumor dynamics, potentially guiding the integration of these combination regimens into clinical practice. The results presented indicate that monitoring volumetric and radiomic features may help clinicians detect early signs of disease progression or resistance, especially relevant when evaluating emerging therapeutic regimens such as PARPi and ICI combinations, even before overt clinical or biochemical recurrence is evident. In particular, the volumetric analysis pinpoints the sites of disease most likely to progress first, enabling more strategic biopsy of suspicious lesions. This, in turn, allows for earlier identification of reversion mutations and other mechanisms of resistance, as well as potential adaptation of therapy (e.g., switching from PARPi to other agents or combining with immunotherapies).

## 5 Conclusion

It is of the upmost importance for clinical trials to understand early response but, although quantitative image-based measurements can provide valuable information about early response, they are in practice not studied routinely. This paper presents a feasibility study showing that noninvasive image-based measurements that complement usual response assessments such as RECIST can be easily extracted, and the results and conclusions presented are applicable to other datasets and trials, ultimately with the potential to improve patient’s safety and outcomes in future trials.

This feasibility study focuses on high-grade serous carcinoma, which is a highly-metastatic, multi-site disease with poor prognosis and limited long-term disease control from systemic anti-cancer treatments. The identification of predictive markers of benefit from ICIs-based combination for a disease with limited response to ICIs like HGSC is very important in clinical practice. However, the multilevel complexities are hindering the ability of standard tools like RECIST to extract meaningful features to develop such predictive markers. Different response assessment measurements were presented in this paper, indicating that total volume changes would be better suited as early response assessment than current assessments by RECIST. Anatomical networks and their number of edges were also introduced as a measurement of spatial heterogeneity and changes thereof, showing differences based on patient response. In general, a decrease in total volume and spatial heterogeneity was found over time, with different metrics being indicative of trends and in some cases significant to evaluate or predict response. These measurements and studies are informative for the analysis of metastatic diseases with high spatial variability in future trials.

## Data Availability

The data analyzed in this study is subject to the following licenses/restrictions: The images analysed in this study were made available from AstraZeneca under a contracted license. More details about how to obtain access to the data underlying the findings in this article in accordance with AstraZeneca’s data sharing policy can be found in 10.1158/1078-0432.CCR-23-2249. Requests to access these datasets should be directed to Details can be found in doi: 10.1158/1078-0432.CCR-23-2249.
