# Supplementary material for: Imaging-based assessment of response to olaparib in platinum-sensitive relapsed ovarian cancer patients
Source: Front Oncol. 2025 Jun 5;15:1546324. doi: 10.3389/fonc.2025.1546324 (PMC12176557; doi:10.3389/fonc.2025.1546324)
Supplement: Supplementary file 1 [file DataSheet1.pdf]

Appendix A Metastatic sites and anatomical distribution

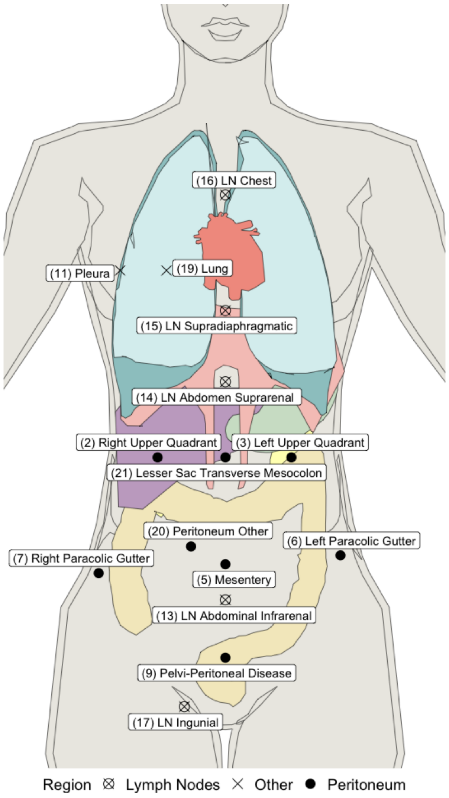

Fig. A1 Illustration created to inform networks conveying information about the complexity of the disease. All sites found in the cohort were set as nodes by using their approximate anatomical locations on a 2D plane.

		Responder at 4 weeks	Enrollment at 1 year	PFS ≥ 9 months	BOR
**Total number of sites**	**t0**	0.3096	0.3199	0.2816	1
	**t1**	0.3519	0.1555	0.2316	0.7693
	**t2**	0.2	0.05094	0.05945	0.2423
**Peritoneal sites**	**t0**	0.3423	0.9376	0.7529	0.7006
	**t1**	0.2258	0.7857	0.6676	0.5249
	**t2**	0.2231	0.3286	0.2234	0.1356
**Lymph nodes sites**	**t0**	0.4432	0.8147	0.9374	0.3484
	**t1**	0.4432	0.8147	0.9374	0.3484
	**t2**	0.186	0.8127	0.4749	0.8631
**Other sites**	**t0**	0.851	0.2548	0.173	0.4074
	**t1**	0.851	0.2548	0.173	0.4074
	**t2**	0.851	0.2548	0.173	0.4074

Table A1 Kruskal-Wallis testing for the number of sites against the different response assessment measurements.

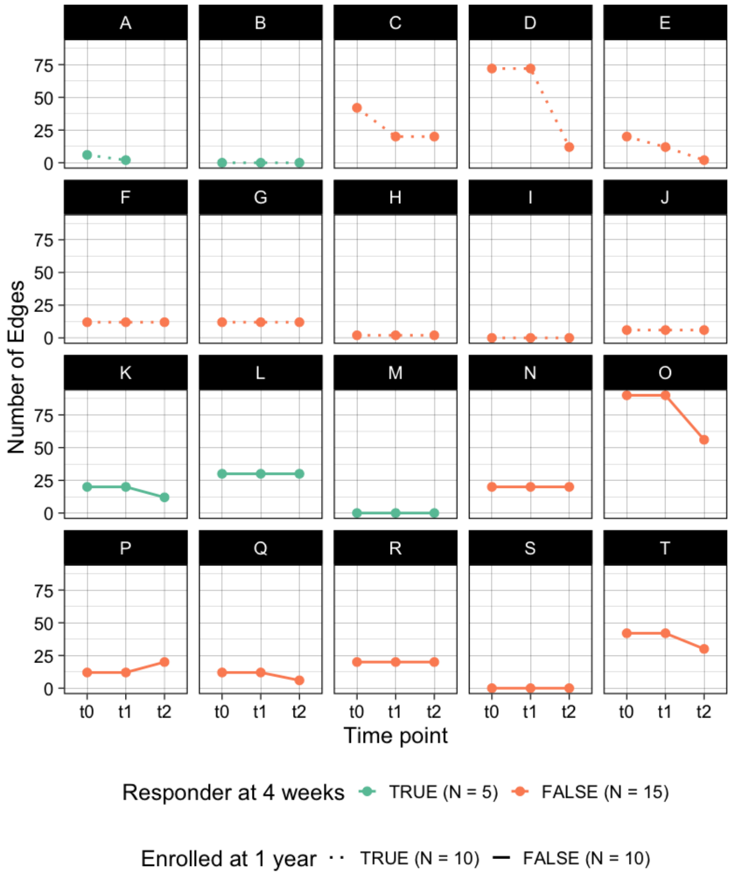

Fig. A2 Number of edges in the anatomical network for every patient and time point.

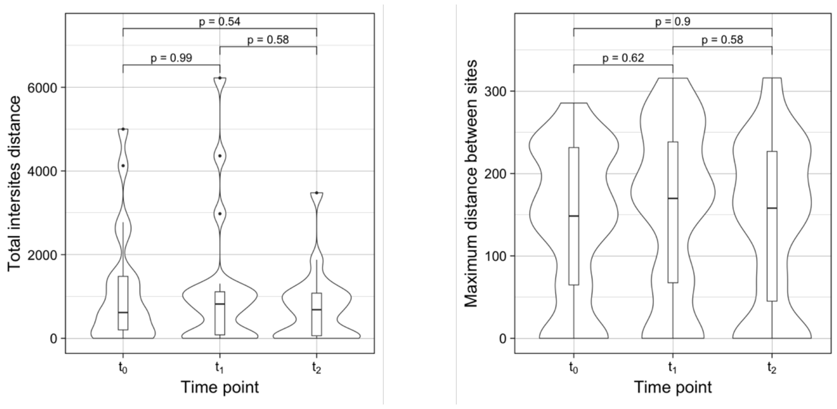

Fig. A3 Temporal evolution of distance dissemination measurements for the whole cohort.

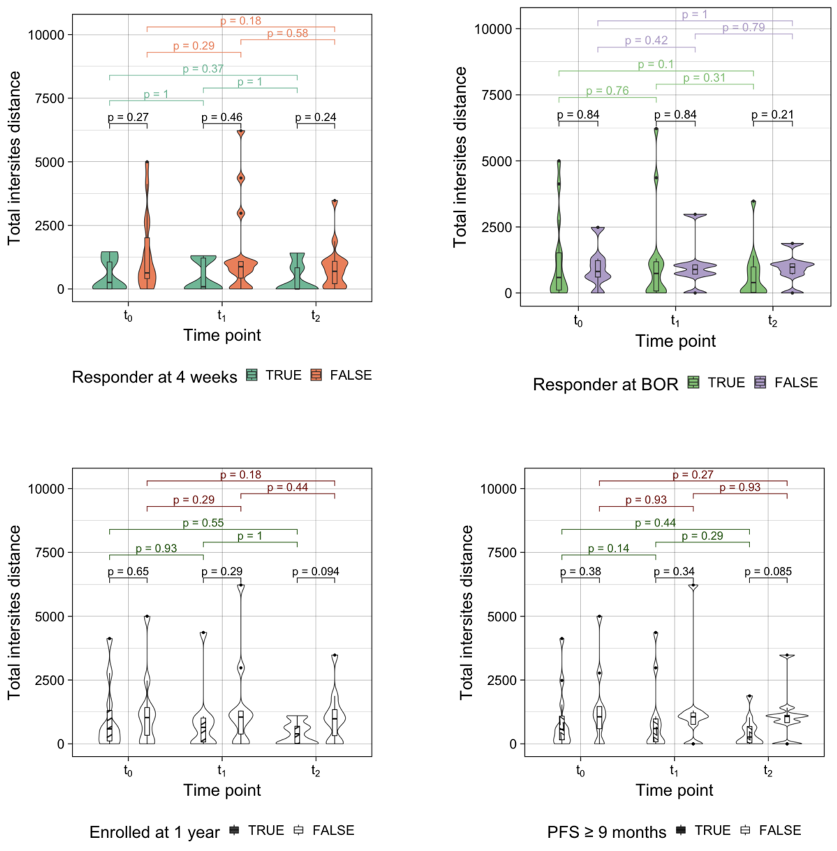

Fig. A4 Comparison of total distance between sites for every response assessment measurement.

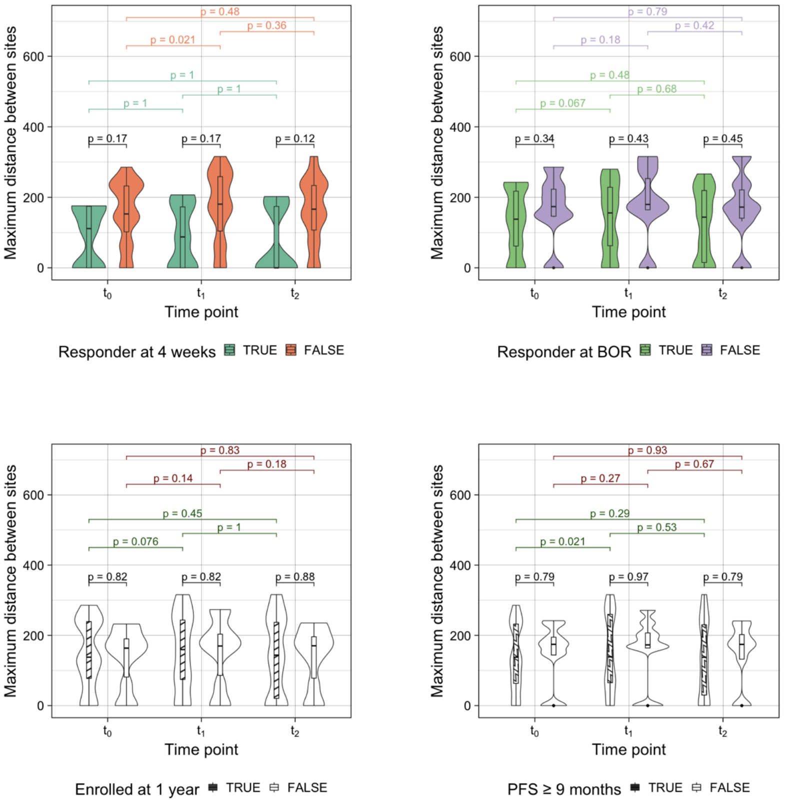

Fig. A5 Comparison of the maximum distance between two sites for every response assessment measurement.

	t0	t1	t2
**RUQ**	3 [1, 7]	3 [1, 6]	3 [1, 5]
**LUQ**	2 [1, 9]	3 [1, 15]	2 [1, 5]
**Mesentery**	5.5 [1, 11]	5 [2, 15]	4 [2, 9]
**LPG**	2.5 [1, 4]	2 [2, 4]	1.5 [1, 2]
**RPG**	2 [2, 3]	2 [1, 15]	2 [2, 6]
**Pelvis**	1 [1, 3]	1 [1, 3]	1 [1, 1]
**Peritoneum other**	2 [1, 3]	2 [1, 3]	3 [1, 3]
**Lesser sac transverse mesocolon**	1 [1, 3]	1 [1, 4]	2 [2, 2]
**Infrarenal abdominal LN**	3 [1, 13]	2 [1, 6]	2 [1, 8]
**Suprarenal abdominal LN**	3 [1, 6]	3 [1, 7]	3.5 [1, 6]
**Supradiaphragmatic LN**	2 [1, 14]	1 [1, 8]	1.5 [1, 8]
**Inguinal LN**	1 [1, 3]	1 [1, 3]	1 [1, 4]
**Chest LN**	1 [1, 11]	2 [1, 14]	3 [1, 11]
**Pleura**	10.5 [7, 14]	11.5 [4, 19]	6.5 [4, 9]
**Lung**	11 [9, 23]	8 [7, 21]	11 [3, 30]

Table A2 Number of lesions per site and timepoint. The numbers correspond to the median and the range.

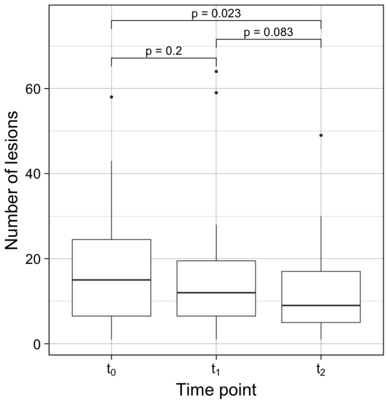

Fig. A6 Number of lesions for the whole patient cohort. Differences between timepoints are assessed through paired sample Wilcoxon signed-rank testing.

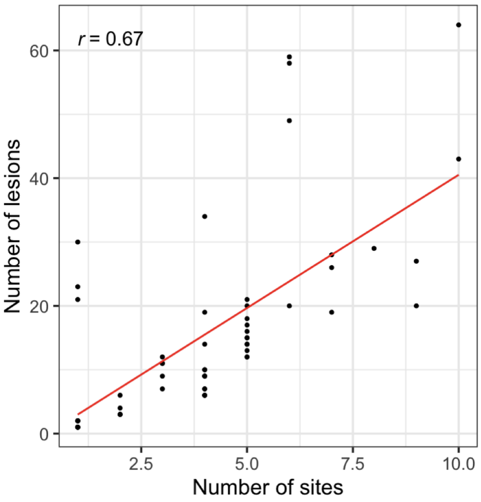

Fig. A7 Pearson correlation between the number of lesions and the number of sites.

3 # Appendix B Volumetric Analyses

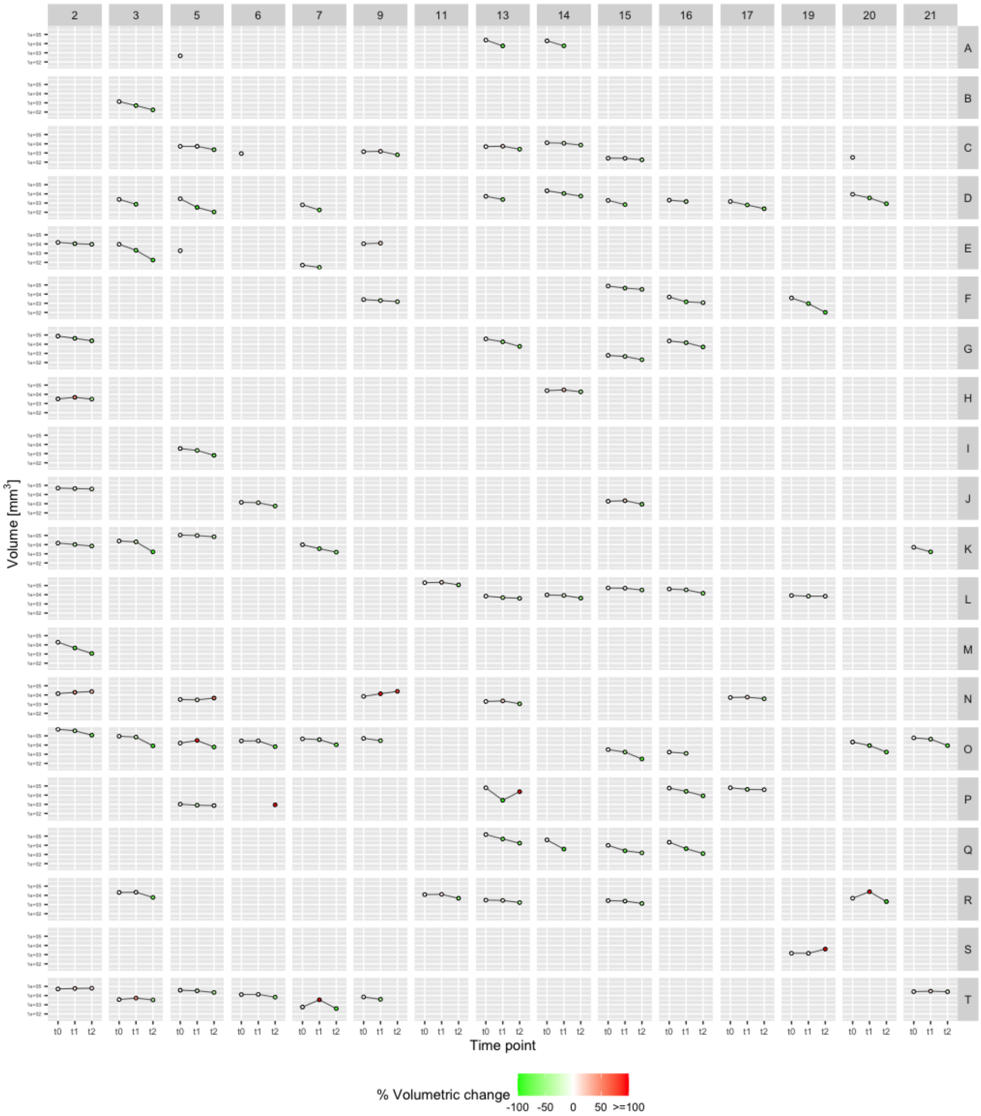

Fig. B1 **Temporal evolution of the volume of every patient individual site in the cohort.**

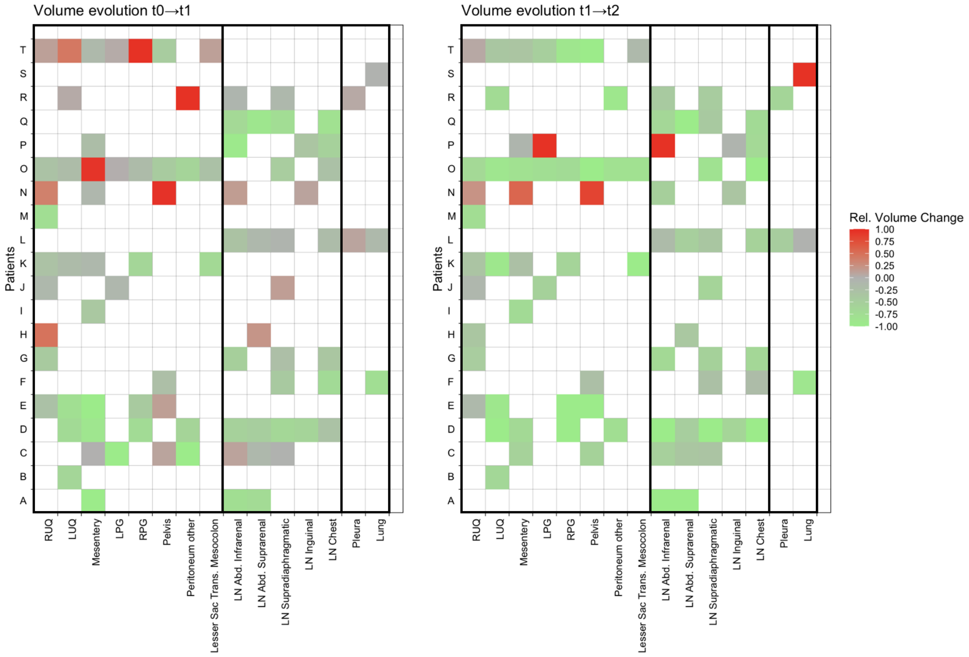

Fig. B2 **Checkers of the volumetric relative site for the sites found in every patient and time point.**

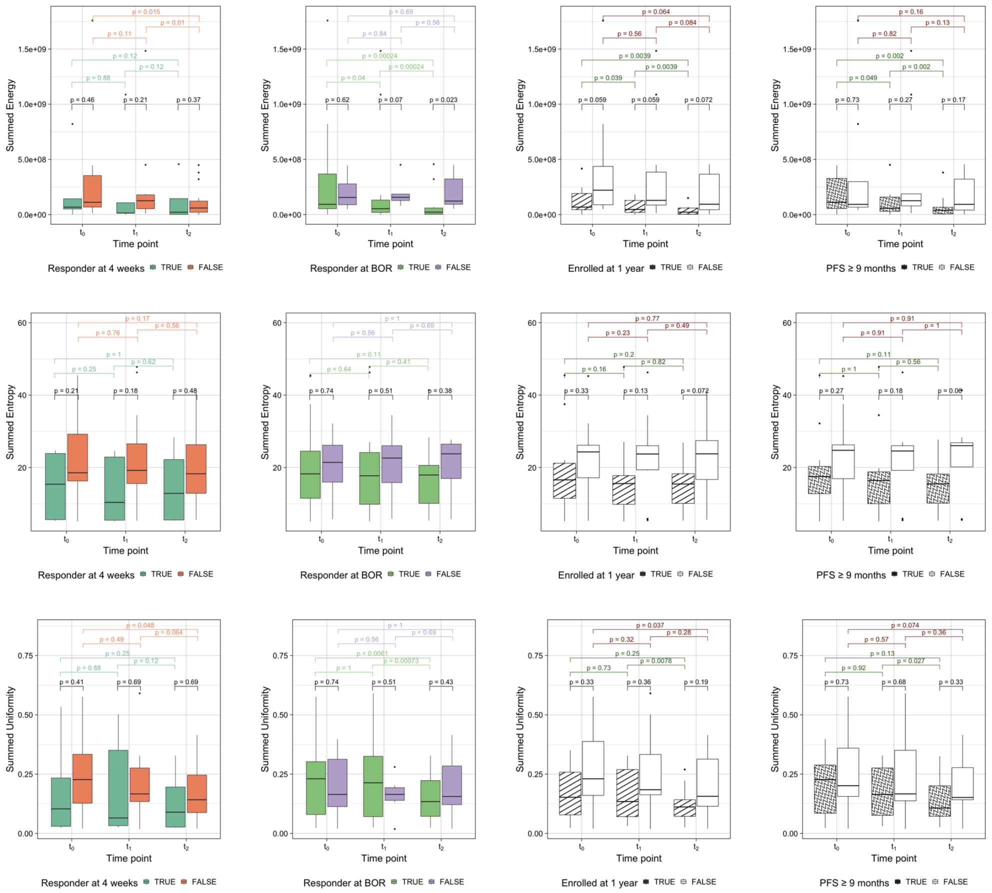

Fig. C1 **Comparison of the overall of summed radiomic features for every response assessment.**

Appendix C Radiomic Analyses

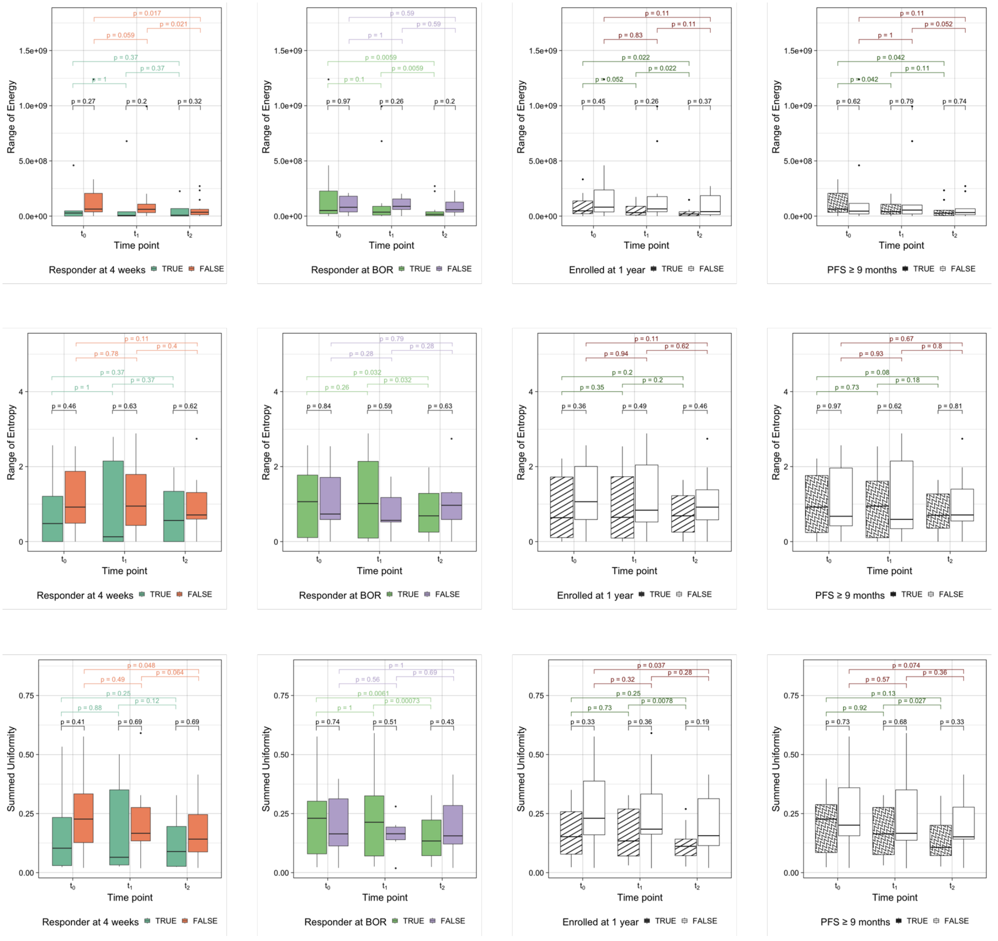

Fig. C2 **Comparison of the range of the radiomic features for every response assessment.**
